# Malignant hyperthermia

**DOI:** 10.1186/1750-1172-2-21

**Published:** 2007-04-24

**Authors:** Henry Rosenberg, Mark Davis, Danielle James, Neil Pollock, Kathryn Stowell

**Affiliations:** 1 Department of Medical Education and Clinical Research, Saint Barnabas Medical Center, Livingston, NJ 07039, USA

## Abstract

Malignant hyperthermia (MH) is a pharmacogenetic disorder of skeletal muscle that presents as a hypermetabolic response to potent volatile anesthetic gases such as halothane, sevoflurane, desflurane and the depolarizing muscle relaxant succinylcholine, and rarely, in humans, to stresses such as vigorous exercise and heat. The incidence of MH reactions ranges from 1:5,000 to 1:50,000–100,000 anesthesias. However, the prevalence of the genetic abnormalities may be as great as one in 3,000 individuals. MH affects humans, certain pig breeds, dogs, horses, and probably other animals. The classic signs of MH include hyperthermia to marked degree, tachycardia, tachypnea, increased carbon dioxide production, increased oxygen consumption, acidosis, muscle rigidity, and rhabdomyolysis, all related to a hypermetabolic response. The syndrome is likely to be fatal if untreated. Early recognition of the signs of MH, specifically elevation of end-expired carbon dioxide, provides the clinical diagnostic clues. In humans the syndrome is inherited in autosomal dominant pattern, while in pigs in autosomal recessive. The pathophysiologic changes of MH are due to uncontrolled rise of myoplasmic calcium, which activates biochemical processes related to muscle activation. Due to ATP depletion, the muscle membrane integrity is compromised leading to hyperkalemia and rhabdomyolysis. In most cases, the syndrome is caused by a defect in the ryanodine receptor. Over 90 mutations have been identified in the *RYR-1 *gene located on chromosome 19q13.1, and at least 25 are causal for MH. Diagnostic testing relies on assessing the *in vitro *contracture response of biopsied muscle to halothane, caffeine, and other drugs. Elucidation of the genetic changes has led to the introduction, on a limited basis so far, of genetic testing for susceptibility to MH. As the sensitivity of genetic testing increases, molecular genetics will be used for identifying those at risk with greater frequency. Dantrolene sodium is a specific antagonist of the pathophysiologic changes of MH and should be available wherever general anesthesia is administered. Thanks to the dramatic progress in understanding the clinical manifestation and pathophysiology of the syndrome, the mortality from MH has dropped from over 80% thirty years ago to less than 5%.

## Disease name and synonyms

Malignant hyperthermia

Malignant hyperpyrexia

## Definition

Malignant hyperthermia (MH) is a hypermetabolic response to potent inhalation agents (such as halothane, sevoflurane, desflurane), the depolarizing muscle relaxant succinylcholine, and rarely, in humans, to stresses such as vigorous exercise and heat. The majority of patients with Central Core Disease (CCD), an inherited myopathy characterized by muscle weakness, are susceptible to MH. Multi-Minicore Disease (MmCD) also predisposes to episodes of MH.

As almost all patients who are MH susceptible have no phenotypic changes without anesthesia, it is impossible to diagnose susceptibility without either the exposure to the "trigger" anesthetics or by specific diagnostic testing. The key diagnostic features include an unexplained elevation of expired carbon dioxide, muscle rigidity and rhabdomyolysis, hyperthermia, acidosis and hyperkalemia.

## Diagnostic criteria

The diagnosis of MH is based on clinical presentation or laboratory testing (see the section on diagnostic methods).

The principal diagnostic features of MH are unexplained elevation of end-tidal carbon dioxide (ETCO_2_) concentration, muscle rigidity, tachycardia, acidosis, hyperthermia, and hyperkalemia. The variability in the order and time of onset of signs often makes the clinical diagnosis rather difficult.

A clinical grading scale was developed by Larach and colleagues [1] in order to assist in clinical diagnosis. The elements of the scale are given in the Table 1. Differential weighting is given to each of the manifestations of the syndrome. However, the scale lacks sensitivity since not all tests may be performed in an individual episode.

**Table 1:** Criteria used in the Clinical Grading Scale for Malignant Hyperthermia

**Clinical Finding**	**Manifestation**
Respiratory acidosis	End-tidal CO_2_>55 mmHg; PaCO_2_>60 mm Hg
Cardiac involvement	Unexplained sinus tachycardia, ventricular tachycardia or ventricular fibrillation
Metabolic acidosis	Base deficit >8 m/Eql pH<7.25
Muscle rigidity	Generalized rigidity; severe masseter muscle rigidity
Muscle breakdown	Serum creatine kinase concentration >20,000/L units; cola colored urine; excess myoglobin in urine or serum; plasma [K+] >6 mEq/L
Temperature increase	Rapidly increasing temperature; T >38.8°C
Other	Rapid reversal of MH signs with dantrolene. Elevated resting serum creatine kinase concentration.
Family history	Consistent with autosomal dominant inheritance

The value of the grading scale is mainly in identifying those subjects with the most convincing episodes of MH for subsequent evaluation of the sensitivity and specificity of the diagnostic tests. The clinical grading scale is useful in evaluating clinical episodes in those cases in which the subject is rated a 6 (almost certainly MH), but lower scores should not be considered for actual diagnosis.

## Epidemiology

The incidence of MH episodes during anesthesia is between 1:5,000 and 1:50,000–100,000 anesthesias. Even though a MH crisis may develop at first exposure to anesthesia with those agents known to trigger an MH episode, on average, patients require three anesthesias before triggering. Reactions develop more frequently in males than females (2:1). All ethnic groups are affected, in all parts of the world. The highest incidence is in young people, with a mean age of all reactions of 18.3 years. It has been found that children under 15 years age comprised 52.1% of all reactions. Although described in the newborn, the earliest reaction confirmed by testing is six months of age [2]. The oldest is 78 years.

Genetically, MH is an autosomal dominant condition; the estimated prevalence of the genetic abnormalities may be as great as one in 3,000 individuals (range 1:3,000 to 1:8,500).

Numerous factors could be involved in triggering MH – age, type of anesthetic, environmental temperature, mitigating drugs administered simultaneously, and degree of stress [3]. Mauritz *et al*. [4] found an incidence of 1:37,500 in patients who had been diagnostically tested, which was similar to the incidence estimated by Robinson *et al*. (1:30,000) [5], although wide variability has been reported. A recent report suggested that the MH susceptible (MHS) trait may be present in 1:2,000–3,000 of the French population [6]. Bachand and colleagues examined the incidence of MH in Quebec, Canada, where many families had been biopsied [7]. They traced the pedigrees of the patients to the original immigrants from France and found an incidence of MH susceptibility of 0.2% in this province. However, that represented only five extended families.

MH crises develop not only in humans but in other species, particularly pigs, which have been a valuable source for research. Reactions have also been described in horses, dogs and other animals [8].

## Clinical description

MH may occur at any time during anesthesia and in the early postoperative period. The earliest signs are tachycardia, rise in end-expired carbon dioxide concentration despite increased minute ventilation, accompanied by muscle rigidity, especially following succinylcholine administration. Body temperature elevation is a dramatic but often late sign of MH. Nevertheless, core temperature should be monitored in all patients undergoing general anesthesia for periods lasting more than 20 minutes, as temperature elevation may be an important confirmatory sign.

Other signs include acidosis, tachypnea and hyperkalemia. The progression of the syndrome may be rapid and dramatic, particularly if precipitated by succinylcholine, or more slower and not become manifest until after several hours of anesthesia.

All inhalation anesthetics except nitrous oxide are triggers for MH. The muscle relaxant succinylcholine is also a trigger for MH. No other anesthetic drugs appear to be triggers, including propofol and ketamine. Neither are catecholamines, nondepolarizing muscle relaxants, catechol congeners, digitalis or similar agents [9].

Although ETCO_2 _is a sensitive early sign of MH, in recent years, with a decline in the use of succinylcholine, rather than an abrupt rise in C02, a more gradual rise is often noted. Indeed, by increasing minute ventilation it is possible to mask this rise [10].

Hyperthermia, when it occurs, is marked by increase in core temperature at a rate of 1–2°C every five minutes. Severe hyperthermia (core temperature greater than 44°C) may occur, and lead to a marked increase in oxygen consumption, carbon dioxide production, widespread vital organ dysfunction, and disseminated intravascular coagulation (DIC) [11].

Uncontrolled hypermetabolism leads to cellular hypoxia that is manifested by a progressive and worsening metabolic acidosis. If untreated, continuing myocyte death and rhabdomyolysis result in life-threatening hyperkalemia; myoglobinuria may lead to acute renal failure. Additional life-threatening complications include DIC, congestive heart failure, bowel ischemia, and compartment syndrome of the limbs secondary to profound muscle swelling, and renal failure from rhabdomyolysis. Indeed, when body temperature exceeds approximately 41°C, DIC is the usual cause of death.

### Succinylcholine induced masseter muscle rigidity

Succinylcholine induced masseter muscle rigidity (MMR) occurs in 1 in 100 children with anesthesia induced by halothane and given succinylcholine [12]. The incidence is probably the same following induction with sevoflurane, but much less following induction with thiopental [13]. The clinical incidence of MH as defined by arterial blood gas changes is about 15% after MMR. However, muscle biopsy reveals that 50% of patients experiencing MMR are MH susceptible [14]. Patients with generalized rigidity along with MMR are at much greater risk for MH. Kaplan (personal communication, 1995) has hypothesized that children with "jaws of steel" after succinylcholine as opposed to mild rigidity are at greater risk for MH. He has hypothesized that the children with the more dramatic masseter rigidity are more often referred for biopsy and hence the high incidence of positive biopsies.

Since MMR may presage MH, it is most advisable to discontinue the anesthetic after MMR. In an emergency, the anesthesia may continue with "non-trigger" drugs. Following MMR, patients should be admitted to an intensive care unit and monitored for signs of MH. Rhabdomyolysis occurs in virtually all patients experiencing MMR and the creatine kinase (CK) values should be checked regularly. Dantrolene should be administered if the other signs of MH occur along with MMR. Muscle biopsy for definitive diagnosis should be carefully considered.

### Central Core Disease and other myopathies

Central Core Disease (CCD) is a rare non progressive myopathy with autosomal dominant inheritance, presenting in infancy and characterized by hypotonia and proximal muscle weakness. A few families demonstrate autosomal recessive inheritance. Histological examination of affected muscles shows a predominance of type I fibres containing clearly defined areas (cores) lacking oxidative enzyme activity [15].

An important feature of CCD is its close association with MH susceptibility. CCD patients are often susceptible to MH by *in vitro *contracture testing (IVCT), but MH and CCD phenotypes do not always co-segregate within families. Patients with MH may present with cores despite being clinically asymptomatic and with some *RYR1 *mutations (specifically some of those in the 3' transmembrane domain of the gene) specific to CCD. A recent study showed that *RYR1 *mutations are found in over 93% (25 out of 27) of Japanese patients with CCD [16]. While this is of importance, it may not reflect the incidence of *RYR1 *mutations in other populations. A more recent study indicated that the distribution and frequency of *RYR1 *mutations differed markedly in the Japanese MH susceptible population as compared to the North American and European MH susceptible population [17]. Although *RYR1 *mutations are the most common identified cause of CCD, it does show genetic heterogeneity, with several rare susceptibility loci known (the *ACTA1 *gene, in association with nemaline myopathy, and the *MYH7 *gene, in association with hypertrophic cardiomyopathy), with further loci yet to be identified [18].

At least 44 mutations have been reported in the *RYR1 *gene in association with CCD (reviewed in [18]). In general terms, single point *RYR1 *mutations can cause (a) CCD only, (b) MH only, (c) MH with variable CCD penetrance. In this latter case, the likelihood of an *RYR1 *mutation resulting in both MH and CCD depends on a number of factors including sensitivity of mutant protein to agonists, size of the intracellular Ca^2+ ^pool and the level of abnormality in channel-gating (reviewed in [19]). All individuals with the mutation should be MH susceptible, while they may or may not have CCD. If a mutation specific to CCD is identified in a family, MH is not automatically excluded as a second mutation may be present and MH susceptibility needs to be assessed by IVCT [20]. If the mutation has no functional studies performed it is of no use clinically. A MH negative parent eliminates susceptibility in the children.

Other myopathies that have been associated with MH susceptibility include some sodium channel forms of myotonia (myotonia fluctuans), MmCD and hypokalemic periodic paralysis [21] and Multiminicore myopathy [22]. Guis *et al*., 2004 found multiminicores in 16 out of 17 MHS patients [23]. The multiminicores correlated with two missense *RYR1 *mutations on the same allele resulting in the amino acid changes R2656W and T2787S [23]. More recently, recessive mutations in *RYR1 *have been associated with MmCD, some of which result in altered Ca^2+ ^release from intracellular stores and others that do not [24]. Taken together, these observations suggest that there may be a subset of *RYR1 *mutations that result in both MH and MmCD and a subset that are associated only with MmCD, similar to the situation with MH and CCD. Consequently, it will be important to distinguish between *RYR1 *mutations that result in MmCD, and those that do not.

King (or King Denborough) syndrome is a rare myopathy characterized by dysmorphic facies, ptosis, down-slanting palpebral fissures, hypertelorism, epicanthic folds, low-set ears, malar hypoplasia, micrognathia, high-arched palate, clinodactyly, palmar simian line, pectus excavatum, winging of the scapulae, lumbar lordosis and mild thoracic scoliosis. The patents with King Denborough syndrome also present congenital hypotonia, slightly delayed motor development, diffuse joint hyperextensibility and mild proximal weakness. Such patients are MH susceptible.

## Etiology

Experimental evidence from a variety of sources, *in vitro*, *in vivo*, isolated cells, transfected cells and mice who's DNA has been altered to express one of the MH causative mutations clearly indicates that the signs and symptoms of MH are related to an uncontrolled release of intracellular calcium from skeletal muscle sarcoplasmic reticulum (SR). In MH susceptible swine and in "knock-in" mice, a variety of environmental conditions can trigger the accelerated calcium release such as environmental heat, exercise and stress. In humans, however, MH results most often from exposure to potent inhalation anesthetics +/- succinylcholine. The enhanced intracellular calcium results in activation of muscle contraction, oxygen consumption, carbon dioxide production, ATP breakdown and heat. The normal sequestration of released calcium is inadequate and energy is expended in a futile manner, in an attempt to lower intracellular calcium. Presumably, the declining levels of ATP lead to failure of membrane integrity and release of potassium and creatine kinase, although the exact steps in the process have not been definitively demonstrated.

In almost all cases, the MH susceptible patients have a defective calcium channel located in the SR membrane. This channel is termed the ryanodine receptor (RYR). The channel is closely associated with other proteins and structures, such as the dihydropyridine calcium channel that mediates transfer of voltage change to the RYR-1 receptor. Other proteins associated with the ryanodine receptor include triadin, and FK 506 binding protein. However, mutations associated with MH susceptibility are found mainly in the gene for the ryanodine receptor. As many as 70% of families susceptible to MH harbor one of about 30 causal mutations for MH, with approximately 40 other mutations that are yet to be characterized [25]. Transfecting cultured muscle cells or myotubes with one of the known causal mutations results in enhanced intracellular calcium release when the cells are exposed to agents such as halothane, caffeine and chlorocresol. Recently, a group has produced a mouse who's genome has been altered to contain one of the causal mutations. The animals and tissues derived from the mouse displayed typical MH changes and raised environmental heat led to typical MH changes [26].

In addition, dantrolene sodium (known to reverse signs of MH) has been found to bind to a specific ryanodine protein site [27].

Although mutations in the ryanodine receptor are undoubtedly important in the pathophysiology of MH, it is also clear that not all families demonstrate linkage to this gene. At least six other genetic loci have been implicated in MH, including one that elaborates the sodium channel [21,28] although only one other gene, *CACNL1A3*, encoding the main subunit of the dihydropyrodine receptor (DHPR), have been shown to be altered by an MH-linked mutation [29].

The clinical expression of MH is also poorly understood. Genotype-phenotype correlations are weak for both the clinical expression of MH and the response of isolated muscle to caffeine or halothane. It therefore seems clear that a variety of modulators influence the manifestations of the syndrome. The fatty acids represent one set of modulators that has been studied in this respect [30,31]. Certain unsaturated fatty acids have been demonstrated to increase the sensitivity of halothane-induced calcium release *in vitro*. Such an increase in fatty acids may result from breakdown of triglycerides as a result of enzymatic abnormalities.

In addition, in cultured muscle cells from MH susceptible patients there is a shift of subtypes of sodium channels leading to a longer membrane depolarization and an increased calcium release from the terminal cisternae [32]. Changes in sodium channel function, either through sodium channel mutations or through effects of fatty acids may influence the phenotypic expression of MH, especially muscle rigidity.

## Laboratory diagnostic methods

The "gold standard" for diagnosis of MH is currently the *in vitro *contracture test (IVCT), which is based on contracture of muscle fibres in the presence of halothane or caffeine. Two widely used forms of this test have been developed; one by the European Malignant Hyperthermia group (EMHG) and the other by the North American Malignant Hyperthermia Group, Caffeine Halothane Contracture Test-CHCT (NAMHG) [33,34]. While similarities exist in performing and interpreting the results of these tests, there are significant differences. Using the EMHG protocol, an individual is considered susceptible to MH (MHS) when both caffeine and halothane test results are positive. A normal MH diagnosis (MHN) is obtained when both tests are negative. A third diagnosis, MH equivocal (MHE), is obtained when only one of the halothane or caffeine test is positive. Using the NAMHG protocol, an individual is diagnosed as MHS when either of the halothane or caffeine test is positive, and MNH when both tests are negative. The EMHG protocol may reduce the possibility of false positive and negative results when compared to the NAMHG protocol but overall similar results are obtained [35]. Sensitivity of 99% and a specificity of 94% are obtained with the EMHG protocol [36], while figures of 97% sensitivity and 78% specificity are reported for the NAMHG. The specificity of either protocol may be affected by neuromuscular disorders unrelated to MH which have an associated increase in myoplasmic calcium concentration. However, studies based on results from monozygote twins indicate that the IVCT has acceptable reproducibility [37]. A third variation of the IVCT, the caffeine skinned fibre test, does not appear to be used diagnostically outside of Japan, and has lower specificity and sensitivity than either the EMGH or NAMHG protocols.

IVCT is expensive, confined to specialized testing centers, it requires a surgical procedure and can yield equivocal as well as false positive and negative results. Modifications of the EMHG protocol include the use of ryanodine [38] (which binds selectively to the calcium release channel) or 4-chloro-m-cresol [39] but to date these agents have not been included in the standard protocol. Future supplies of halothane may be limited. A possible alternative testing agent is the fluorinated ether, sevoflurane. Trials with this agent are to be commenced soon. Other biochemical, hematological and physical tests have been used in the past but, without exception, these lack significant sensitivity and specificity to be used diagnostically.

DNA analysis, however, offers an alternative to the IVCT, requiring only a blood specimen, which can be sent to an accredited diagnostic laboratory. DNA testing for MH was first suggested in 1990, when a mutation within the ryanodine receptor gene (*RYR1*) encoding the skeletal muscle calcium release channel was identified [40]. Since then, about 50% of MH have been linked to *RYR1 *with over 100 mutations associated with MH, identified within this gene [41].

MH is, however, a heterogeneous genetic disorder with at least five other susceptibility loci being identified. Amongst these loci, mutations with a clear association with MH have been identified in only one gene, *CACNA1S*, encoding the alpha subunit of the dihydropyridine receptor, the voltage sensor of the skeletal muscle calcium release channel [29,42,43]. Early studies suggested that mutations cluster within three regions of *RYR1 *(reviewed in [19]) largely because many laboratories screen only these regions for the presence of mutations in MH susceptible patients. Complete screening of the entire coding regions of *RYR1 *has, however, revealed that mutations occur in almost all regions of the gene [17,41].

While the majority of mutations lead to a single amino acid change in the receptor [44], deletions or truncations have also been reported [45-47]. A number of recessive mutations have also been reported to result in either MH or CCD [48].

The identification of causative mutations suggests the widespread use of DNA testing for MH, however, this is confounded by the metabolic complexity and genetic heterogeneity of the disorder. Moreover, discordance between MHS diagnosed by IVCT and the presence of causative mutations has been shown within individual families [49-55]. In most cases, genotype-phenotype discordance where MHS has been diagnosed by IVCT is likely to be due to the presence of a separate mutation either in *RYR1 *or at a separate locus [6,56]. False positive results for the IVCT cannot be ruled out however, and probably account for a small proportion of such discordance. False positive results could be expected as the IVCT is not 100% specific and could be due to an unrelated myopathic condition. Until a greater understanding of the pathophysiology of these other conditions and their relationship to the MH phenotype is known, these patients would need to be treated as MHS. Discordance in patients with a causative mutation but who have been diagnosed MHN by IVCT has also been reported by some testing laboratories [55]. As causative mutations clearly show altered calcium release from intracellular stores, these rare instances of discordance are likely to be due to a false negative diagnosis in the IVCT. Taken together, these observations suggest that DNA testing should always be used in selected, genetically characterized families, as well as within the guidelines for DNA testing identified by the EMHG [57] or the NAMHG [58]. Using both IVCT and genetic diagnosis, a higher proportion of true positives are likely to be identified than by simply relying on one or other test.

The guidelines set down by the EMHG requires contracture testing prior to genetic testing in a family. Once a causative mutation is found in a family member, others may be tested for susceptibility by seeking out that mutation in the DNA. The predictive value of the genetic test has been suggested to be ~50% based on correlation between an MHS diagnosis by IVCT and presence of a causative mutation [58]. Using the same criteria, 62% of MHS patients in one family (36/58) were diagnosed MHS by genetic testing [56]. Another study reported the predictive value of genetic testing to be ~80% in a total of 10 families [55]. The sensitivity of the DNA test clearly varies within populations but will likely increase as more mutations are identified especially for those families where a significant level of discordance has been observed.

In summary, because of the heterogeneity of the disorder, as well as discordance within families, a negative DNA result cannot be used to rule out MH susceptibility. In addition, only those mutations that have been biochemically characterized to affect SR calcium release can be used to test for MH susceptibility. Approximately 28 mutations within *RYR1 *have been shown to cause an alteration in calcium release from intracellular stores. A number of functional tests have been used successfully to assess the role of *RYR1 *mutations in calcium release. These include the use of lymphoblastoid cell lines generated from MHS individuals [59-61]. COS-7 or HEK293 cells transfected with the cDNA for rabbit *RYR1 *carrying point mutations introduced by site-directed mutagenesis [19,62,63], myotubes generated from muscle biopsy tissue [44,64-66] and 1B5 dyspedic myotubes transduced with wild type and mutated *RYR1 *cDNA [67]. Calcium release can be monitored and quantified using calcium-specific indicators like fluo-4 and fura-2 [68], [^3^H] ryanodine binding assays [67,69], and, indirectly, by protein release [70]. Systems using 1B5 dyspedic myotubes are more physiological as they constitutively express all the components of the skeletal muscle with the exception of *RYR1 *[67]. They also contain larger and more efficiency filled calcium stores than do COS-7 or HEK 293 cells, thus providing a more sensitive measure for calcium release and reloading. To date, all mutations functionally characterized have been shown to cause alterations in calcium flux through the ryanodine receptor calcium release channel. It has been argued that lymphoblastoid cells and myotubes derived from MH patients can not be used to unambiguously demonstrate altered physiological function, as the phenotype may be due not only to mutations in *RYR1 *but also in other genes encoding protein components of the SR calcium release channel. Nevertheless, these systems have proved useful in demonstrating abnormal calcium release associated with *RYR1 *mutations and it could be equally argued that these systems are representative of the genetic background of individual patients and therefore provide valuable information *ex vivo*. As lymphoblastoid cell lines do not express the dihydropyridine receptor, they could also effectively be used to functionally test *RYR1 *mutations as well as to eliminate *RYR1 *as a causative factor in MH individuals who do not show linkage to *RYR1*.

A variety of minimally invasive diagnostic tests are in development at present. One utilizes nuclear magnetic resonance spectroscopy to evaluate ATP depletion during graded exercise *in vivo*. MH patients have a greater breakdown of ATP and creatine phosphate, as well as an increase in acid content compared to controls [38]. The test requires expensive and sophisticated equipment and a team well versed in interpreting the resultant tracings of the peaks of ATP and inorganic phosphate. Insertion of a microdialysis catheter into muscle and injection of a small amount of caffeine will elicit an enhanced release of carbon dioxide from the muscle tissue, which can be measured by capnography [71].

## Differential diagnosis

A variety of unusual conditions may resemble MH during anesthesia. These include sepsis, thyroid storm, pheochromocytoma, and iatrogenic overheating. Hence, a high index of suspicion for these disorders as well as the ability to measure ETCO_2 _and obtain arterial and venous blood gas analysis is essential in order to differentiate MH from these disorders. Particularly problematic is the unexplained hyperthermia following anesthesia. Since anesthetic gases generally inhibit the febrile response, the first sign of sepsis may be marked hyperthermia on emergence from anesthesia. Response to antipyretics as well the clinical setting is often helpful in differentiating this response from MH.

The differential diagnosis of unexplained increased ETCO_2 _includes hyperthermia secondary to sepsis, or iatrogenic warming, machine valve malfunction, rebreathing, as well as faulty equipment.

Outside the operating room, MH-like syndrome may occur following injection of ionic contrast agents into the cerebrospinal fluid, cocaine overdose, and the neuroleptmalignant syndrome(NMS).

NMS is a potentially fatal hyperthermic syndrome that occurs as a result of ingestion of drugs used in the treatment of mental and nervous conditions such as schizophrenia. The incidence is approximately 0.01–0.02% of those being treated with these drugs such as older as well as newer antipsychotics and haloperidol, a sedative agent often used in the ICU to treat agitation. Other dopamine antagonists also have been reported to cause NMS.

The signs of NMS include muscle rigidity, acidosis, high fever, rhabdomyolysis. The pathophysiology is thought to result from dopamine receptor blockade. Treatment includes benzodiazepines, bromocriptine and even dantrolene. There does not appear to be any cross over susceptibility to MH or *vice versa*. There is no laboratory diagnostic test for the syndrome either [72].

If a high ionic, water-soluble radiologic contrast agent is injected intrathecally, usually as a result of drug mixup, a characteristic progression of signs occurs. After the injection, the patient appears to recover normally, but within thirty minutes involuntary jerking movements begin in the lower extremities and ascend to the upper body, finally resulting in seizures and hyperthermia. This is the result of the contrast agent entering the cerebral ventricles and requires a rapid symptomatic treatment of the muscle activity, hyperthermia, and acidosis (cooling, nondepolarizing neuromuscular blockers, ventilation, and sedation) [73]. The response of signs of hyperthermia, tachycardia and tachypnea to dantrolene in such syndromes is non-specific. In other words, the response to dantrolene does not *per se *prove MH susceptibility.

In many countries, a "hotline" has been established to provide emergency assistance in the management of MH. Many are listed on the web site of the Malignant Hyperthermia Association of the US [74].

### Hyperkalemic cardiac arrest in patients with muscular dystrophy

A syndrome often confused with MH is sudden hyperkalemic cardiac arrest during or shortly after anesthesia in young males. Following sporadic reports of such arrests, Larach and colleagues identified that patients with an occult myopathy, especially a dystrophinopathy such as Duchenne's muscular dystrophy, are at risk to dramatic life-threatening hyperkalemia upon administration of succinylcholine [75]. More recently, it has been shown that administration of potent volatile agents to such patients may produce a similar syndrome [76].

Since the most common muscular dystrophy (Duchenne's) is found with a frequency of 1 in 3500 live male births, and the onset of symptoms of muscle weakness may be as late as 6–8 years of age, some apparently healthy children may really be at risk of succinylcholine induced hyperkalemia. Hence, when a young child or young adult experience a sudden and apparently unexpected cardiac arrest, think of hyperkalemia, document and treat it in the standard fashion (calcium, bicarbonate, glucose and insulin, and hyperventilation). Muscle tissue should be obtained and preserved for testing for a myopathy, specifically a dystrophinopathy.

In general, the patient with a dystrophinopathy that develops these anesthetic-related complications does not also exhibit classic signs of MH, such as hypethermia or marked muscle rigidity. They do, however, develop rhabdomyolysis. Therefore, this reaction is not malignant hypethermia *per se*, since the dystrophinopathies are caused by mutations on the X chromosome.

In response to the presentation of over 30 such cases to the Food and Drug Agency (FDA) in 1992, a warning was issued to avoid the use of the drug in children and young adolescents for elective cases. Succinylcholine should be reserved for those cases of full stomach and possibly airway related emergencies.

### Rhabdomyolysis

Rhabdomyolysis refers to the breakdown of skeletal muscle which is associated with excretion of myoglobin in the urine. Classically, MH presents with hypercarbia, tachycardia, cardiac arrhythmias, pyrexia, rigidity and metabolic acidosis, and rhabdomyolysis as a late sign. Several reports of isolated rhabdomyolysis apparent immediately following anesthesia or developing up to 24 hours post anesthesia have been published [77,78]. Increased creatine kinase (CK) measurement and a positive IVCT have been obtained in these patients, indicating MH susceptibility. However, MH-like muscle responses can represent false positive diagnoses and an underlying myopathic process may produce a positive IVCT [79], so there must remain some doubt on the validity of this feature *i.e*. rhabdomyolysis as an expression of MH. Burns *et al*. however stated that MH should be considered in all patients presenting with rhabdomyolysis where the degree of muscle necrosis exceeds that expected for the severity of the accompanying disorder [80]. The most prudent diagnostic course, therefore, is contracture testing for MH susceptibility.

## Genetic counseling

Genetic testing can be defined as an analysis or test that confirms the presence or the absence of a genetic condition; this does not necessarily involve the analysis of DNA as there are still many clearly genetic conditions where the gene has not yet been identified. In the context of MH, the IVCT could be considered to be just as much a genetic test as the analysis of the *RYR1 *gene.

Genetic testing is different to the traditional medical test in that not only will the result have potential ramifications for the current health of that individual, but it may also have ramifications for the future health of that individual and the future health of their immediate relatives [81,82]. Depending on the test being performed, results may leave the individuals disadvantaged in terms of their ability to access health insurance or life insurance, employment opportunities and, in some cultures, may even affect marital opportunities [83,84]. For this reason it is recommended that each individual accessing any form of genetic testing and indeed each individual undergoing IVCT or *RYR1 *analysis should be fully informed of all the implications of each potential result and should be able to provide informed consent [85,86]. The process of imparting this information and discussing any questions the patient may have, is known as genetic counseling. This discussion with a clinician or genetic counselor should include the following points of information (Am. Soc. Hum. Gen. 1975):

- Potential implications the result may have on their ability to obtain health/life insurance [87,88].

- Potential psychological effects of the result. Some parents feel guilty that they may have passed MH sensitivity on to their children, others may feel anxious about the implications of a MH sensitivity result and experience an increased fear of surgery, others may even feel guilty if they have not inherited MH when their brother or sister has [89-91].

- Inheritance pattern of the disorder and what implications their test result may have for their children and the extended family.

If there is also a family history of CCD, it is important that the potential diagnosis of this condition is not lost in the discussions regarding MH. CCD is an extremely variable condition within families and while some individuals may only be very mildly affected, other family members may have a more severe phenotype [15].

### Interpreting risk for other family members

MH is an autosomal dominant condition. When initiating genetic analysis in a branch of a known family, it is important to test the individual at the highest risk first.

#### Parents of a proband

The large majority of times, an affected proband will have inherited MH sensitivity from one of the parents. Clarification of which parent may also be MHS is useful for identifying which side of the extended family (*i.e*. aunts and uncles) may be at risk.

#### Siblings of a proband

The risk to the siblings depends of the genetic status of the parents. If a parent is identified as MHS, then each of the proband's siblings have a 1 in 2 or 50% chance of also being MHS. If both parents receive an MHN result on IVCT and *RYR1 *analysis – suggesting the mutation is *de novo *in the proband – then the proband's siblings are at no greater risk than the general population.

#### Offspring of a proband

The risk for offspring of each individual with proven MHS also has a 50% chance of being MHS. The proband's grandchildren would be considered to be at 25% risk until their parent's genetic status is clarified.

Note: An individual who is MHN cannot pass MH sensitivity on to the next generation, however, if they have an affected parent, their siblings may still be at risk.

### Interpretation of risk for other family members in the context of RYR1 analysis

As discussed in earlier sections, the identification of a causative *RYR1 *mutation is sufficient to diagnose MH sensitivity. However, due to current concerns regarding discordance between IVCT and mutation analysis in some families, current protocols state that a negative mutation result is not sufficient to identify a person as being MHN. In the event of a normal (negative) mutation result, IVCT is still recommended to confirm MHN status, and the individual and his/her offspring are still considered to be potentially MHS unless IVCT proves otherwise [54].

However, it is important to remember that in the event of a normal (negative) mutation result, the offspring of the individual are no longer at risk of inheriting the characterized family mutation. As the tested individual does not carry the mutation, he cannot pass it on to his offspring. Therefore, if an individual is mutation negative but IVCT positive, the only useful test available to the offspring is IVCT.

### Final note on autonomy in clinical testing for MH

Some individuals may wish to delay IVCT or *RYR1 *analysis, while they consider the information they have been given and/or make the necessary preparations. Others may decide that they do not want their risk clarified by clinical testing. These decisions should be respected and these individuals considered being MHS until proven otherwise.

Care should then be taken when arranging testing for the offspring of these individuals as a positive result in the next generation will generate a result for the individual who did not want to know (the individual must have carried the gene mutation in order to pass it on).

## Management and treatment

### Acute MH crisis

The essential points in the treatment of acute MH crisis are the immediate discontinuation of trigger agents, hyperventilation, administration of dantrolene in doses of 2.5 mg/kg repeated prn (*pro re nata*) to limit MH, cooling by all routes available (especially nasogastric lavage), and treating hyperkalemia in a standard fashion. Calcium blockers should not be used along with dantrolene, since hyperkalemia may occur with such a drug combination. The steps in the treatment of acute MH are as follows:

1. Stop potent inhalation agents and succinylcholine.

2. Increase minute ventilation to lower ETCO2.

3. Get help.

4. Prepare and administer dantrolene:

- 2.5 mg/kg initial dose;

- Titrate dantrolene to tachycardia and hypercarbia;

- 10 mg/kg suggested upper limit, but more may be given as needed.

5. Begin cooling measures:

- If hyperthermic, use iced solutions, *i.e*. Ice Packs to groin, axilla, and neck;

- Nasogastric lavage with iced solution;

- More aggressive measures as needed;

- Stop cooling measures at 38.5°C.

6. Treat arrhythmias as needed. Do not use calcium channel blockers.

7. Secure blood gases, electrolytes, creatine kinase, blood and urine for myoglobin;

- Coagulation profile check values every 6–12 hours;

- Treat hyperkalemia with hyperventilation, glucose and insulin as needed;

- Once crisis is under control, an MH hotline should be contacted for further guidance.

8. Continue dantrolene at 1 mg/kg every 4–8 hours for 24–48 hours.

9. Insure urine output of 2 ml/kg/hour with mannitol, furosemide, and fluids as needed.

10. Evaluate need for invasive monitoring and continued mechanical ventilation.

11. Observe patient in Intensive Care Unit for at least 36 hours.

12. Refer patient and family to MH Testing Center for contracture or DNA testing.

Patients experiencing MH should receive dantrolene and be monitored closely for 48–72 hours, since (even despite dantrolene treatment) 25% of patients will experience a recrudescence of the syndrome. Tests for disseminated intravascular coagulation (DIC) should be included, as well as observation of the urine for myoglobinuric renal failure. DIC is most frequent when body temperature exceeds about 41°C.

### Preventive measures

Preventive measures include a thorough anesthetic history to determine the possibility of the patient or a family member having experienced an MH episode. When suspicion of MH exists, family members should not be given trigger anesthetic agents, *i.e*. potent volatile anesthetic agents such as halothane, sevoflurane, desflurane, enflurane, isoflurane and succinylcholine, and testing is recommended.

Patients with any form of myotonia should not receive succinylcholine. Patients with hypokalemic periodic paralysis, CCD, Duchenne or Becker muscular dystrophy, paramyotonia, or myotonia fluctuans should not receive trigger agents.

All patients receiving more than a brief general anesthetic should have their core temperature monitored.

Young patients (below age 12 approximately) should not receive succinylcholine for elective procedures, in order to avoid the possibility of hyperkalemic response in a patient with undiagnosed muscular dystrophy.

Patients who are MH susceptible should be cautioned regarding the remote, but conceivable possibility of heat stroke in environments in which exposure to high heat and humidity is possible.

### Management of the MH susceptible for anesthesia

Patients who are known to be MH susceptible may be anesthetized with regional anesthesia or local anesthesia without problems. If general anesthesia or sedation is required, the potent volatile agents and succinylcholine should be avoided.

The anesthesia machine should be prepared by flowing 100% oxygen through the machine at 10 L/min for at least 20 minutes. The ventilator should also be included in purging the machine by cycling the ventilator at the time of the oxygen flow. Vaporizers should be disabled, drained or removed if possible. All intravenous agents and nondepolarizing relaxants are safe to use.

Once the patient has undergone such an anesthetic without incident, he/she may be treated similar to any other patients. It is no longer felt to be necessary to monitor such patients in the post-anesthesia care unit for four hours routinely. Pretreatment with dantrolene is also not necessary.

## Unresolved issues

### Discordance

Given the confidence provided by functional analysis of *RYR1 *mutations, the problem of discordance between *RYR1 *mutations and MHS and MH equivocal (MHE) diagnosis still remains the largest problem associated with genetic diagnosis of susceptibility to MH. The MHE diagnosis is the most problematic and exhibits a much higher level of discordance than does MHS. Correlation between *RYR1 *mutations and IVCT is greater for the caffeine (c) than the halothane (h) response [92] suggesting that the MHE(c) has greater diagnostic potential. The NAMHG protocol does not allow the MHE diagnosis; the potential for discordance between IVCT phenotype and *RYR1 *genotype is therefore much greater. In a large UK study investigating the relationship between *RYR1 *genotype and IVCT phenotype, discordance was identified in seven families (nine individuals), with five false-positives and four false-negatives [20]. Mutation negative MHS individuals have also been observed [20,55] (Recent unpublished data give an approximate 2.5% discordance rate of this type in a large series of UK patients). Clear evidence of the involvement of genes as well as *RYR*, has been shown in a New Zealand Maori pedigree where MHS correlates with a T4826I mutation [56]; but three branches of the family possess unrelated chromosome 19 haplotypes, without the T4826I mutation in unambiguous MHS individuals spanning three or four generations. While some discordance may be explained by the existence of other yet unidentified mutations, false positive IVCT tests [93] and weak contracture mutations [20] have also been implicated. Clearly, while genetic diagnosis can be used selectively, a greater knowledge of the molecular mechanisms resulting in susceptibility to MH is required before the IVCT can be dispensed with.

### Awake MH

In 1966 the Porcine Stress Syndrome was identified as an "awake" malignant hyperthermia episode. Stresses such as fighting cause a rapid death in these animals. In 1974 Wingard described an MH susceptible family with exercise and emotional induced pyrexia, in sudden deaths unrelated to surgery. He considered that MH was part of a human stress syndrome. Subsequently, a number of reports of MH reactions in patients given trigger-free anesthetics appeared. None of these reactions were totally convincing.

However, Gronert and Denborough, both reported patients with "awake" MH episodes, the latter being patients with exercise-induced heat stroke who responded to dantrolene [94-96]. Perhaps the most convincing, though unfortunate, episode of exercise induced MH was reported by Tobin *et al*., a fatal episode in a 13-year-old boy who had experienced a clinical episode of MH and developed signs of MH following exercise some months later. He and other family members were found to have a causative *RYR 1 *mutation [97]. Brown *et al*. reported a possible viral trigger [56].

Further physiological evidence of stress-related MH has been demonstrated by pH changes in MHS muscle recovering from violent exercise [98]. The sympathetic nervous system appears to be only secondarily involved [99], but serotonin (5HT) agonists may cause an MH-like syndrome in susceptible pigs [100]. These agents can also cause MHS contractures in susceptible muscle [101]. Does serotonin have a role in the stress-induced episodes? There is limited support for this [102,103]. Recent research in mice with the Y522S mutation indicates abnormal sensitivity to increased environmental temperatures associated with abnormal calcium release. This latest report, however, should be considered with some caution as the homozygous Y522S mutation in mouse is embryonic lethal, which is a different phenotype to that observed with the homozygous R615C mutation in pigs and the small number of homozygous *RYR1 *mutations in humans which clearly do not cause embryonic lethality. A more recent study, however, reports that a "knock-in" mouse heterozygous for the R163C *RYR1 *mutation is more representative of the human phenotype and thus may provide an important model system for further study of awake-MH [104].

Wappler *et al*. described a 34-year-old male with recurrent fever, fatigue, muscle cramping, and aching with mild exercise and emotional stress [105]. IVCT demonstrated an MHS response and a "causative" mutation. Others have reported similar findings [106,107]. A possible conclusion is that a small subset of MH patients may display muscle damage and perhaps more ominous signs with the stress of exercise, and may be other stresses. It is recommended that MH is excluded in patients who have had episodes of exert ional heat stroke [108]. Despite possible links between exertional heat stroke and MH however, treatment with dantrolene has had limited results, thus this drug should not be used routinely in the management of heat stroke.

## Resources

Many anesthesia textbooks, web sites and articles contain very thorough descriptions of MH and related syndromes. However, these sources often fail to provide information for patients (patient-specific information). Various voluntary organizations throughout the world are dedicated to assisting patients, physicians, anesthesia providers of all types and any one else in managing the MHS and keeping these individuals up to date with the latest information regarding MH.

In the United States, the Malignant Hyperthermia Association of the United States (MHAUS) provides newsletters, printed information, an informative website [75] to meet the needs of the various groups interested in MH. In addition, a hotline provides direct consultation for providers in real time management of MH episodes or questions related to specific patient as to their likelihood of developing MH and the optimum management of an episode. MHAUS, similar to other MH patient advocacy organizations is not for profit supported by voluntary contributions. The North American MH Registry supports a patient-specific database with detailed information as to the phenotypic presentations as well as diagnostic test results. The Registry is a subsidiary of MHAUS and is located at Children's Hospital of Pittsburgh [109].

The European MH group [110] coordinates testing procedures throughout Europe and is made up of professionals investigating MH. Patient supported MH associations exist in France, Germany, Switzerland, Japan, United Kingdom and several other countries. In South Africa, issues related to MH are subsumed under the Muscular Dystrophy Association of that country. These organizations have been crucial to the education of anesthesia providers in diagnosing and managing MH and helping patients better understand the disorder.
